# Can adopting skin cancer preventive behaviors among seafarers be increased via a theory-based mobile phone-based text message intervention? A randomized clinical trial

**DOI:** 10.1186/s12889-020-09893-x

**Published:** 2021-01-14

**Authors:** Esmat Heydari, Tahereh Dehdari, Mahnaz Solhi

**Affiliations:** Department of Health Education and Health Promotion, Faculty of Public Health, Iran University of Medical Sciences, Shahid Hemmat Highway, Tehran, Iran

**Keywords:** Protection motivation theory, Skin cancer, Text message, Seafarer, Intervention study

## Abstract

**Background:**

One of the main occupational hazards for seafarers is the long exposure to sunlight. This study aimed to determine the efficacy of a mobile phone-based text message intervention in adopting skin cancer preventive behaviors among a sample of seafarers in Genaveh port located in Bushehr province, Iran.

**Methods:**

In this randomized controlled trial, 136 seafarers were randomly selected and assigned to the intervention (*n* = 68) or a control groups (*n =* 68). As a theoretical basis, we followed the Protection Motivation Theory (PMT) variables to develop the text messages. The data related to PMT variables and skin cancer preventive behaviors were collected through a questionnaire. Forty-five text messages were designed, pre-tested and sent to the seafarers’ phones in the intervention group in 45 days. Both groups were followed up 1 month after the intervention. Data collected in the two stages were analyzed using paired-samples *t*-test, ANCOVA, and Chi-square tests.

**Results:**

Following the intervention, the mean scores of adopting skin cancer preventive behaviors (*p* = 0.001), perceived self-efficacy (*p* = 0.01), protection motivation (*p* = 0.02), and fear (*p =* 0.001) were significantly higher in the intervention group than the control group. There was significant reduction in the response costs (*p* = 0.05) and perceived rewards (*p* = 0.01) scores in the intervention group compared with the control group after the intervention. However, there were no significant differences in the perceived vulnerability (*p* = 0.14), perceived severity (*p* = 0.09), and response efficacy (*p* = 0.64) between the two groups after the intervention.

**Conclusions:**

The results of the study indicated the effectiveness of mobile phone-based text message intervention for increasing skin cancer preventive behaviors in Iranian seafarers.

**Trial registration:**

Iranian Registry for Clinical Trial (the link to trial: https://www.irct.ir/trial/7572). Registered 16 July, 2016. Prospectively registered.

**Supplementary Information:**

The online version contains supplementary material available at 10.1186/s12889-020-09893-x.

## Background

Skin cancer is considered as one of the serious public health problems which has affected millions of people worldwide. In many countries, the prevalence of the cancer has been increased in recent years [1]. The results of a study in Iran showed that the incidence of basal cell carcinoma, squamous cell carcinoma and malignant melanoma has had an increasing trend in recent years. For example, the number of registered cases of skin cancer increased from 7320 in 2004 to 9964 in 2008 in this country [2].

A combination of factors such as increased exposure to ultraviolet (UV) or sunlight, increased outdoor activities, changes in clothing style, increased longevity, ozone depletion, genetics, and immune suppression is probably the cause of the rising incidence rate of this type of cancer [3].

Solar radiation exposure is a health risk in several groups of workers employed in outdoor occupations permanently such as those who work at sea (e.g. fisherman and seafarers). These workers are identified as the group at risk for the development of skin cancer [4–6]. Feister et al. reported that UV index values at tropical and subtropical oceans could exceed UVI = 20, which was more than twice that of typical mid-latitude UV index values [7]. Modenese et al. found that the potential individual UV exposure of the fishermen was between 65 and 542 J/m2. The percentages of the ambient exposure were estimated between 2.5 and 65.3% [6].

Despite the prevalence of skin cancer among seafarers [8], many of them are not aware of the risks of the ultraviolet radiation in the sea; some of them enjoy sunbathing a lot [9]; also, a large number of them do not protect themselves against the sunlight exposure at sea [5].

Given the importance of adopting sun-protective practices (e.g. wearing long sleeves, hats and sunscreens; avoiding direct exposure to sunlight between 10 am and 4 pm; and limiting exposure to UV light) in the prevention of skin cancer [1, 10], more attention should be paid to holding educational programs through various communication channels to encourage the seafarers to perform sun protective behaviors [5, 11].

One of the communication channels is the short message service (SMS) text-messaging system. The effectiveness of developed interventions based on this channel has been revealed in such studies [12, 13]. Furthermore, using behavioral change theories is recommended to hold educational interventions. The theories may help us to better understand a specific problem in a particular context and attempt to explain the behavior determinants for developing tailored interventions [14, 15].

In the present study, the Protection Motivation Theory (PMT) variables were considered as the conceptual framework for developing text messages. This theoretical framework has been used to develop interventions in the field of skin cancer prevention [1]. The theory was proposed by Rogers in 1975 [14]. Protection motivation is originated in two basic components: (i) threat appraisal and (ii) coping appraisal. The threat appraisal assesses the individuals’ perceptions of the severity of and their susceptibility to the threat. The coping appraisal consists of the individuals’ belief that the recommended behavior will affect the reduction of the threat (i.e. response efficacy) and the idea that the individual is able to perform the recommended behavior (i.e. perceived self-efficacy) [16].

Given the importance of adopting sun-protective practices in the prevention of skin cancer in seafarers [5] and the advantages of developing mobile phone-based text message interventions in the behavior change [13], we conducted the present study.

### Aim of the study

The overall goal of the study was to determine the efficacy of a mobile phone-based text message intervention in adopting skin cancer preventive behaviors. As a theoretical basis, the researchers followed the PMT variables for developing text messages.

## Methods

### Design and hypotheses

A randomized controlled trial (parallel type) was conducted in order to determine the effect of a mobile phone-based text message intervention in adopting skin cancer preventive behaviors among a sample of Iranian seafarers. Our main hypothesis was that the mobile phone-based text message intervention would significantly increase the skin cancer preventive behaviors in the intervention group from pre-test to post-test compared to the control group. The study participants were assessed at baseline and at 1-month follow-up.

### Setting

The participants were recruited from Genaveh port during August to December, 2016.

### Study location

Genaveh port lies in Bushehr province, south-western Iran. Due to staple sunlight angle and clean atmosphere, Bushehr province has a high intake of sunlight radiation on its surface throughout the year. The results of a study showed that during 2012, there were 208 days (56.83%) with UV index 8–10 and 52 years (14.21%) having UV index 6–7 in this province. The mean of UV index per month from March to September in the province has been reported 10. In addition, the mean of UV index per month from October to March varied between 4 and 8 in the province. As to intake of solar energy, Bushehr province is considered a high radiation area [17, 18]. Literature review showed that skin cancer had a high incidence rate in the southern provinces of Iran, such as Bushehr province [19].

### Participants

A total of 136 seafarers were recruited. Trial eligibility criteria were: (i) having willingness and consent to participate in the study, (ii) being fluent in the Persian language, (iii) being seafarers at least for 1 year, (iv) currently residing in the trial area and expecting to be resident for the duration of the trial (v), owning or having, daily access to a cellphone and (vi) having no history of skin cancer. Since only men are employed in seafarer occupation in the port of Genaveh, the study participants were male seafarers. At the first visit, written informed consent was obtained if the participants met all the inclusion criteria.

### Randomization

After the baseline assessment was completed, an independent researcher using a web-based randomization program (Sealed Envelope.com) randomly allocated the participants (*n =* 136) to either the intervention or control groups (no intervention) at a ratio of 1:1. The researcher involved in the primary outcome assessment remained blind to the allocated treatment group. Due to the nature of the intervention, the participants were not blind to their allocation. A “Welcome” text message was sent to the cell phone of the participants in the intervention group.

### The SMS-intervention program development

First, according to the pre-test results regarding PMT variables and skin-cancer preventive behaviors, a total of 45 text messages were designed in Persian. Then, the developed text messages were pre-tested on 15 seafarers (apart from the study participants) in three focus group discussion. After explaining the purpose of the meeting, each message was written on the board and they were asked to discuss the following 10 questions about each message: 1. Who is the target population of this message?, 2. Can you easily understand the concept of the message?, 3. Did the message ask you to do something specific? If yes, What?, 4. Was there anything in the message that you do not believe in? What?, 5. Is there anything in the message that makes you annoyed or ridiculed?, 6. What do you remember about this message?, 7. Did the message contain a specific notion that you do not like? What?, 8. Did the message contain a specific notion that you like? What?, 9. Was the message containing confusing content?, 10. Do you have any suggestions to make this message better?, and 11. Which message did you like the most? Why? [20]. The responses and comments of the participants were written and then applied in the messages. Finally, according to their suggestions, two messages were edited. The final text messages (*n =* 45) are shown in Table 1. The time and frequency of sending the text messages were mutually agreed by the researcher and the seafarers. Seafarers preferred to receive one message a day at 4.00 pm. For a period of 45 days, one member of the research team sent one of the developed messages (Table 1) daily at a time (4 p.m.) to the participants’ cell phone. Within the first 21 days, the messages focused on perceived vulnerability, perceived severity, and perceived self-efficacy. In days 22–45, the messages focused on response costs, response efficacy, perceived rewards, fear, protection motivation and sun-protective practices. In order to rectify the possible failure of the system, delivery reports of the mobile phones were checked by one of the researchers of the present study.

**Table 1:** SMS messages adopted following the focus group discussions

Variables	Messages
Perceived vulnerability	1. Do you know that skin cancer is a common cancer among seafarers? 2. Do you know that skin cancer is the most common type of cancer in many parts of our country (Iran)? 3. Remember that direct exposure to sunlight is one of the factors predisposing the seafarers to skin cancer.
Perceived severity	4. Dear Seafarer: Skin cancer is a serious condition. It may be fatal. 5. Although there is no definitive cure for the skin cancer, it is preventable. 6. Have you ever had sunburn? Have you ever wondered what would be the side-effects of the repeated sunburn on your skin?
Perceived self-efficacy	7. Health is the chief asset to man. Protect yourself against the skin cancer. 8. If you will, you can adopt preventive behaviors to prevent skin cancer. 9. It may be a difficult task for you to buy sunscreen. You can get help from your wife or your sister to buy it. 10. Don’t be lazy. In case of sweating, swimming, or washing your hands or face, the re-use of sunscreen is essential. 11. Despite the heat, if you want, you may reduce the exposure of your body skin to sunlight while working outdoors by wearing long-sleeved blouse and long pants. 12. It is not so difficult to purchase a cap. It is inexpensive and can be found in most shops. 13. Some people are lazy or unwilling to wear a cap. If health matters to you, make a serious decision to regularly wear a cap. 14. Although it may be difficult and costly for you to buy standard sunglasses, if you value your health, purchase one and use it. 15. Even if others make fun of you, if you want, you can still wear sunglasses with a large screen that covers around your eyes well when exposed to the sunlight.
Response costs	16. You might say that my job is to always work in the sunlight, and I cannot prevent the sunlight from impacting my body. This may be a bit difficult, but it is very possible. Thus, give it a try. 17. Although buying and using a cap and sunglasses may be difficult for you, with regular use, you may protect yourself from the sun rays. 18. Purchasing foreign sunscreens can be expensive and costly; however, you can buy and use good quality Iranian sunscreen at a much lower price. 19. Some people may think you are wearing sunglasses for beauty or style and always make fun of you. Explain to them why you are using these things. You may even persuade them to use these things as well. 20. Although wearing long sleeves clothes and long pants will make you feel warmer, keep in mind that doing so reduces your skin exposure to the sunlight. 21. Although when you wear a cap, you sweat and feel warm in your hair, you protect the skin of your head against the sun rays. 22. Although buying standard and medical sunglasses that prevent the sun ultraviolet costs a lot, you can buy and use one by a little savings. 23. Although you may worry a little by getting information about skin cancer and its protective measures, remember that it is a good feeling and guarantees your health.
Response efficacy	24. Health is the chief asset to man. If you want, you may protect yourself against skin cancer. 25. By adopting precautionary measures (in subsequent SMSs), you may reduce the probability of developing skin cancer in yourself and minimize your concerns. Thus, follow our next SMSs.
Perceived rewards	26. By protecting yourself from the sunlight, you may prevent other medical problems such as cataract besides the prevention of skin cancer.
Fear	27. Many people get scared and worried when they think about skin cancer. This fear is natural, which may be counteracted by reducing the exposure to the sunlight. 28. The fear of skin cancer is a common feeling in most people. This fear may act as a trigger to adopting sun-protective behaviors.
Protection motivation	29. Decide right now and protect your skin more than ever before. 30. Although your job is as such to spend most of the day outside, make a decision today and reduce your exposure to the sunlight. 31. Decide today to wear a cap, sunglasses, and sunscreen when exposed to the sunlight. 32. Decide from now to go outside less or get in the shade when the sun has the most radiation during the day (11 AM to 5 PM). 33. See your doctor as soon as possible by observing abnormal nevi and spots on your skin.
Sun-protective practices	34. The amount of ultraviolet ray in the sunlight is very high from 11 AM to 5 PM. Try as much as possible not to expose yourself to the sunlight in unnecessary situations to prevent the skin cancer. If you have to do work under the sunlight, try to be in the shade. 35. There is ultraviolet radiation from the sun even in winter or cloudy weather. Thus, reduce your unnecessary activities between 11 AM and 5 PM as much as possible. 36. Being in the shade (especially between 11 AM and 5 PM) is great and pleasant for anyone. The shade may reduce up to 50% of the intensity of the sun ultraviolet radiation. 37. To reduce the exposure to the sunlight, apply sunscreen 20 min before you leave the house. 38. It is best to re-apply your sunscreen every 2 h. Even when you wash your face or sweat, you should use the sunscreen again. 39. The skin around the eyes has a great potential for skin cancer. Wear standard sunglasses even on cloudy days. 40. Buy your sunglasses from reliable stores, and after buying them, make sure that they are of a medical type using the devices available in eyeglasses stores. 41. Using gloves (preferably dark) may reduce the exposure of your skin to the sunlight. 42. Wearing long-sleeved blouses and long pants (preferably dark) when working outdoors reduces the amount of sun UV rays exposure to your body skin. 43. Wearing a cap (preferably dark) or making a shade reduces the exposure of your face, ears, and neck to the sunlight. 44. Check your entire skin every month. If you see any new nevi, moles, nevi with irregular margin, asymmetric nevi, non-uniform colored nevi, large, itchy, or painful nevi, wounds that are bleeding and do not heal and red masses or spots, see your doctor immediately. 45. See your doctor once every 6 months to examine your skin. Accelerate your treatment and healing process by the early diagnosis of your skin problems.

### Procedures to promote the intervention fidelity and reduce the between-group contamination

The seafarers were asked not to share the text messages with others. One of the researchers of the present study was in charge of recording and describing any contact between the participants and the research team.

### Measures

At baseline assessment, the participants^,^ demographic characteristics including age, education level, marital status, parents’ education level and the history of sunburn in the past month were collected.

#### Primary outcome

The primary outcome of the study was the change in the mean score of skin cancer preventive behaviors from baseline to follow-up. The secondary outcome was the change in the mean scores of the PMT variables from baseline to follow-up. These outcomes were measured using a validated instrument by Morowatisharifabad et al. for assessing the determinants of *skin cancer* preventive behaviors *among* Kazeroon *farmers* using PMT variables [21]. It is noteworthy that permission to use and publish the instrument in the current study was obtained orally from him. The instrument previously has not been published elsewhere. The instrument measured PMT variables in terms of skin cancer prevention and sun protective behaviors among Iranian farmers [21]. Items of the instrument used are shown in Additional file 1. The validity of the items of the instrument was measured using face validity and the qualitative content validity. To do so, a panel including 20 experts in health education and dermatologists studied the items and reflected on the simplicity, clarity and readability, grammar, wording, scoring, and relevance of the items. According to their comments, some unclear questions and minor wording errors were corrected. The internal consistency of the sub-scales was measured by Cronbach’s α. To this end, 20 farmers completed the instrument. Cronbach’s α of the perceived vulnerability, perceived severity, response efficacy, fear, perceived self-efficacy, response costs, perceived rewards, protection motivation (or behavioral intention), and adopting skin cancer preventive behaviors subscales were reported as 0.81, 0.70, 0.84, 0.79, 0.86, 0.80, 0.73, 0.78 and 0.85, respectively [21].

### Trial procedures

The duration of the interventions was 45 days, with a follow-up period of one moth.

#### Baseline data collection

**Fig. 1:**
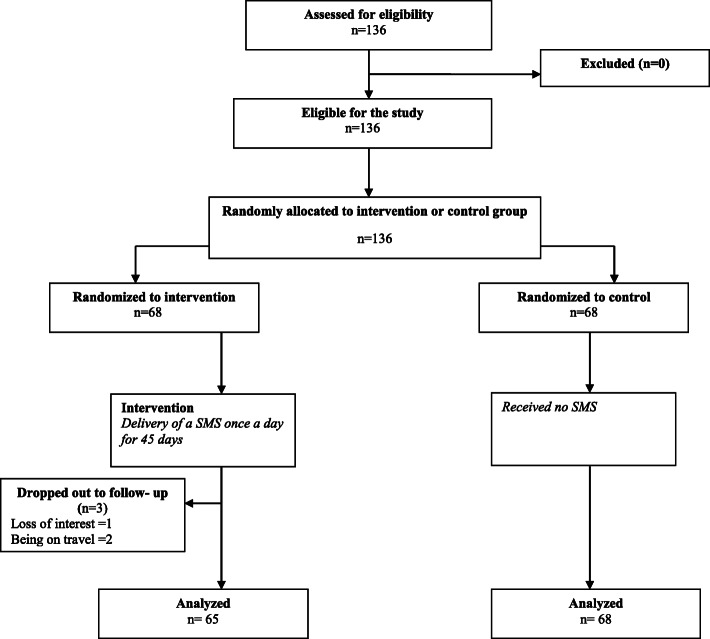
Follow-up of study participants

A list of the seafarers’ names in the Port and Maritime Organization of the Islamic Republic of Iran in Genaveh port was generated (*n* = 1000). Based on the list, one thousand random numbers were generated using R software (version 3.6.0). The first 136 seafarers of the list were selected. One of the researchers contacted with the seafarers^’^ phone number and informed them about the study objectives. All the participants who were contacted initially (*n* = 136) agreed to participate in the study and fulfilled the eligibility criteria (Fig. 1). The decision about determining the time of completing the instrument was made with the seafarers’ consent. The instrument was filled out in a private room at the maritime health center of Genaveh port. Eligible participants were provided with the information about the trial and, if willing, entered the study. The participants were free to ask the researcher their questions about the study. After obtaining written informed consent of the seafarers and providing them with detailed contact information including the participant’s cell number, the data were registered in the cell phone of one of the trial researchers. Then, the instruments were completed by the participants.

#### Follow up to 1 month

The participants were followed 1 month after the education delivered via text messages and completed the instrument again to determine the changes in adopting skin cancer preventive behaviors and PMT variables. The seafarers who missed their scheduled visit for 1 month were actively followed by one of the research staff and their reasons were obtained.

### Sample size calculations

The formula “*n =* (Z _1-α/2_+Z_1-β_)^2^. (S_1_^2^+S_2_^2^)/d^2^” was used to calculate the sample size. Input data to estimate the needed sample size came from the findings of a pilot study that had been conducted by the present trial researchers. Given that the perceived severity was recognized as a main predictor of adopting sun-safe practices in seafarers [5], standard deviations of the variable of both intervention and control groups at 1 month (15.67 and 21.35) were used. A total of 136 seafarers (*n* = 68 per group) provided sufficient statistical power (> 80%) with an alpha of 0.05 (2-sided) to detect the difference of 10 scores in the change in the skin cancer preventive behaviors at 1 month from the baseline compared with the control group over the 1-month study interval, allowing for up to 25% loss to follow up.

### Statistical analyses

The homogeneity of baseline data in demographic characteristics of the intervention and control groups was determined by χ^2^. The distribution (or normality) of PMT variables and adopting skin cancer preventive behaviors was tested by Kolmogorov–Smirnov test. Since the data were normally distributed, differences in PMT variables and adopting skin cancer preventive behaviors between, before and after the intervention in each group were tested using Student’s paired-samples t test. In addition, differences in PMT variables and adopting skin cancer preventive behaviors between the two groups were also tested using Analysis of Covariance (ANCOVA). In the study, data are expressed as means and standard deviations and all statistical tests were considered significant at the level of 0.05.

### Ethics approval

This trial was approved by the Research Ethics Committee at Iran University of Medical Sciences (Code: IR.IUMS.1395.28113). A written informed consent was obtained from the participants. This submission adhered to CONSORT guidelines.

## Results

Between August 2016 and September 2016, the study participants were selected and randomized. The follow-up for the last recruited participant was completed in December 2016. There was also 2.21% loss to follow up. Three participants in the intervention group were excluded due to personal reasons including having no interest (0.73%) and being on travel (1.47%).

Table 3 summarizes the demographic characteristics of the participants in the two groups. The results of Chi-Square test showed that there was no statistically significant difference between both groups for any of the demographic, PMT and skin cancer preventive behaviors variables before the intervention (Table 2).

**Table 2:** Descriptive statistics (means (SD) and percentages) of the participant characteristics in the intervention (*n* = 65) and the control group (*n* = 68)

Variables	Intervention group	Control group	***p***-value ^1^
	***N*** (%)	***N*** (%)	
**Age**			0.85
≤ 30 years old	20 (30.8)	19 (27.9)	
31–40	21 (32.3)	25 (36.8)	
41–60	24 (36.9)	24 (35.3)	
**Mothers` education level**			0.16
Illiterate	43 (66.2)	56 (82.4)	
≤ 12th (grade)	22 (33.8)	10 (14.5)	
> 12th (grade)	0 (0)	2 (3)	
**Father` education level**			0.53
Illiterate	34 (52.3)	42 (61.8)	
≤ 12th (grade)	23 (35.4)	23 (33.9)	
> 12th (grade)	8 (12.3)	3 (4.4)	
**Marital status**			0.56
Single	18 (27.7)	19 (27.9)	
Married	47 (72.3)	49 (72.1)	
**Education level**			0.63
≤ 12th (grade)	42 (64.6)	49 (72.1)	
> 12th (grade)	23 (35.4)	19 (27.9)	
**History of sunburn in the past month**			0.43
Yes	20 (30.8)	19 (27.9)	
No	45 (69.2)	49 (72.1)	

^1^ Results of χ^2^ for the comparison of differences in demographic variables between the two groups

Following the intervention, the mean scores of adopting skin cancer preventive behaviors, perceived self-efficacy, protection motivation and fear scores were significantly higher in the intervention group than the control group (Table 3). There were significant reductions in response costs and perceived rewards scores in the intervention group compared with the control group after the intervention. In addition, there were no significant differences in the perceived vulnerability, perceived severity, and response efficacy scores between the groups after the intervention (Table 3).

**Table 3:** Comparison of the PMT variables and adopting skin cancer preventive behaviors before and after the intervention in the intervention and the control group

Variables	Intervention group		*p*-value*	Control group		*p*-value*	*p*-value**
	Before intervention	After intervention		Before Intervention	After intervention		
Perceived vulnerability	25.80 ± 3.41	27.53 ± 3.04	0.002	25.67 ± 2.82	26.75 ± 3.02	0.01	0.14
Perceived severity	24.24 ± 4.03	27.80 ± 6.74	0.001	24.29 ± 3.37	26.22 ± 3.77	0.001	0.09
Response costs	32.40 ± 6.15	26.76 ± 7.47	< 0.0001	29.00 ± 5.83	29.57 ± 6.82	0.54	0.05
Response efficacy	18.21 ± 4.12	20.83 ± 3.37	< 0.0001	18.13 ± 3.32	20.55 ± 3.21	< 0.0001	0.64
Fear	11.95 ± 0.27	14.98 ± 3.5	< 0.0001	11.87 ± 0.62	12.18 ± 0.42	0.001	0.001
Perceived self-efficacy	35.93 ± 9.89	42.24 ± 5.47	< 0.0001	37.10 ± 7.01	40.00 ± 5.86	0.002	0.01
Perceived rewards	11.87 ± 3.56	9.35 ± 2.78	< 0.0001	12.02 ± 3.27	11.13 ± 4.91	0.20	0.01
Protection motivation (or behavioral intention)	19.66 ± 4.84	21.33 ± 4.15	0.01	19.27 ± 4.88	19.72 ± 4.12	0.49	0.02
Adopting skin cancer preventive behaviors	17.26 ± 5.79	21.18 ± 4.66	< 0.0001	17.23 ± 6.30	18.58 ± 3.99	0.07	0.001

Values are Mean ± SD.

^*^ Result of the Student paired-samples t test (within-groups comparison)

**Result of the Analysis of Covariance (between-groups comparison)

*p* ≤ 0.05 significant

The comparison of pre- and post-test results in the intervention group showed that there was a significant increase in the perceived vulnerability, perceived severity, response efficacy, fear, perceived self-efficacy, protection motivation (or behavioral intention), and adopting skin cancer preventive behaviors scores. In addition, the response costs and perceived rewards scores showed a significant reduction in the intervention group compared with before the intervention (Table 3).

The comparison of pre- and post-test results in the control group showed that there were significant increases in the perceived vulnerability, perceived severity, response efficacy, fear, and perceived self-efficacy scores. In addition, there were no significant differences in perceived rewards, response costs, protection motivation (or behavioral intention), and adopting skin cancer preventive behaviors scores in the control group compared with before the intervention (Table 3).

## Discussion

The findings of this study showed that adopting skin cancer preventive behaviors among the participants in the intervention group significantly increased after the mobile phone-based text message intervention compared with the control group. This finding is in line with similar studies which showed that use of mobile phone-based interventions can be an effective strategy used to improve cancer prevention behaviors [12, 22, 23]. Further use of mobile phone technology for changing various dimensions of unhealthy lifestyle of seafarers is suggested in Iran.

The findings also showed that three variables including perceived vulnerability, perceived severity and response efficacy did not show significant differences in the intervention group compared with the control group after the intervention. The findings are consistent with the findings of some studies [22, 24, 25]. Of course, several studies have shown that the education intervention had significant effects on these variables [1, 25–27]. Probably, one of the reasons for the obtained results was that before the onset of the study, most of the participants in the two groups believed that skin cancer is a serious and fatal disease; everyone may be vulnerable to the disease and adopting sun-protective behaviors may decrease the probability of the risk of the disease in the future. In other words, the participants in both groups had good information regarding skin cancer and its preventive methods before the intervention. Lack of sun-protective behaviors (despite high awareness) among the participants may be related to their high perceived barriers for adopting the behavior, low self-efficacy beliefs and so on. Therefore, the identification of the important predictors of adopting skin cancer prevention behaviors and performing interventions to address the variables is suggested.

In the present study, following the intervention, the mean scores of the perceived self-efficacy, fear, and protection motivation (or behavioral intention) variables significantly increased in the intervention group compared to the control group. The findings are also consistent with, some previous studies [1, 28, 29]. For example, Anderson et al. found that implementing computer-based interventions can improve the perceived self-efficacy of food suppliers for consumption of fat, fiber, fruits, and vegetables [29]. Given the importance of the variables predicting cancer prevention behaviors [1, 30, 31], it is recommended that more attention should be paid to those variables. These theoretical variables can assist in better understanding of the cancer prevention behavior and developing effective efforts.

The results also showed that the mean score of the response costs variable considerably decreased in the intervention group compared with the control group after the intervention. This finding is consistent with those of some similar studies [1, 25, 26, 28]. Response costs in PMT act as barriers in adopting the recommended behavior [1]. Literature also showed that there were many barriers to performing skin cancer prevention behaviors [32, 33]. Reducing or removing the barriers may encourage the individual to perform healthy behaviors [1, 34]. It is noteworthy that in the study, eight messages regarding response costs associated with adopting skin cancer preventive behaviors were developed. These messages tried to provide practical and simple suggestions for the participants to reduce the barriers of the behavior. For example, a message was designed with this content that “Instead of the foreign expensive sunscreens, you can use Iranian sunscreens at a more reasonable price”. These messages could reduce the perceived barriers of adopting sun protective behaviors (such as high prices of sunscreens) and facilitate the behavior among the participants.

In the present study, a significant decrease in the perceived rewards variable was reported in the intervention group compared to the control group after the intervention. This finding is in line with those of Babazadeh et al. [1] and McClendon et al. [35]. The finding showed that the perceived rewards of long exposure to sunlight such as being delightful in cold weather decreased among the participants in the intervention group. Providing more information about the rewards of adopting sun-protection behavior would be encouraging for the seafarers to get more involved in the process of reducing long exposure to the sunlight.

The important point of our study is that it was the first theory-based cell phone intervention in the cancer prevention field among seafarers. The findings of this study can be useful for health educators and occupational health professionals as well. The limitations of this study were the short follow-up period. In addition, in the study, data were obtained based on a self-report questionnaire, which might cause probable bias in the results.

## Conclusions

The results of the study indicated the effectiveness of mobile phone-based text message intervention for increasing skin cancer preventive behaviors in Iranian seafarers. Given that this intervention proved to be effective, it could be applied for other groups of workers employed in outdoor occupations such as farmers, fisherman and traffic police officers who have long exposure to sunlight.

## Supplementary Information

**Additional file 1.** Items of instrument used

## Data Availability

The datasets used and/or analysed during the current study are available from the corresponding author on reasonable request. Please contact the corresponding author for the data requests.

## Abbreviations

SMS: Short-message service
PMT: Protection motivation theory
UV: Ultraviolet
