# Supplementary material for: Can adopting skin cancer preventive behaviors among seafarers be increased via a theory-based mobile phone-based text message intervention? A randomized clinical trial
Source: BMC Public Health. 2021 Jan 14;21:134. doi: 10.1186/s12889-020-09893-x (PMC7807693; doi:10.1186/s12889-020-09893-x)
Supplement: Supplementary file 1 — Additional file 1. Items of instrument used [file 12889_2020_9893_MOESM1_ESM.docx]

Instrument used to measure the protection motivation theory in relation to skin cancer preventive behaviors (developed by Morowatisharifabad et al [16])

| **Variable** | **Item** | **Responses (coding used for analyses** |
| --- | --- | --- |
| **Perceived vulnerability** | 1. It is likely that I catch skin cancer.  2. Those who are under the sun for long hours are more likely to catch skin cancer.  3. I am not so much exposed to the sun daily that I catch skin cancer.  4. Skin cancer is seen in those with white skin.  5. If I do not use a cap under the sunlight, I am more likely to catch skin cancer.  6. If I do not use sunscreen when I am under the sun, it is more likely that I get afflicted with skin cancer.  7. In my local area nobody is afflicted with skin cancer, so I will not catch the disease either.  8. Only those with skin cancer in the family will catch the disease | 8 items on a 5-point scale (1= strongly disagree; 5= strongly agree) |
| **Perceived severity** | 1. Skin cancer is a very dangerous disease.  2. If skin cancer is not diagnosed early, it will lead to death.  3. Skin cancer is not that worrying.  4. Skin cancer is easily diagnosed and treated.  5. The cost of skin diseases is very high and will cause financial problems for me.  6. Affliction with cancer will disturb my social life.  7. Affliction with cancer will greatly affect my job. | 7 items on a 5-point scale (1= strongly disagree; 5= strongly agree) |
| **Fear** | 1. I have a bad feeling regarding skin cancer.  2. When I think about skin cancer, I get worried that I might get the disease.  3. When I am exposed to the sun for a long time, I am frightened I get skin cancer.  4. I am worried since I have not still taken any measures as to prevention of skin cancer.  5. Thinking about death after skin cancer frightens me. | 5 items on a 5-point scale (1= strongly disagree; 5= strongly agree) |
| **Response costs** | 1. It is time-consuming to get some information about skin cancer.  2. Using sunglasses and cap is troublesome for me.  3. Sunscreen is expensive.  4. I sweat when I use sunscreen.  5. If I wear sunglasses, my friends make fun of me.  6. I feel hot when I wear long-sleeved shirt.  7. I feel hot and my hair sweats when I wear a cap.  8. Sunglasses are too expensive to buy.  9. When I do some protective measures against skin cancer, I get more worried about the disease. | 9 items on a 5-point scale (1= strongly disagree; 5= strongly agree) |
| **Response efficacy** | 1. If I reduce my exposure to sun, it is less probable to catch skin cancer.  2. If I wear a cap, it is less probable to catch skin cancer.  3. If I wear sunglasses, it is less probable to catch skin cancer.  4. Using sunscreen has a great role in prevention of skin cancer.  5. When I take some protective measures against the sun (wearing caps, sunglasses, sunscreen, etc.), I am no more worried about catching skin cancer. | 5 items on a 5-point scale (1= strongly disagree; 5= strongly agree) |
| **Perceived rewards** | 1. It is a pleasure for me to rest and be exposed to the sun.  2. I have a good feeling when I am exposed to the sun.  3. Sunray can make my skin healthier.  4. When I do not wear a cap, I do not feel hot since I am exposed to the air. | 4 items on a 5-point scale (1= strongly disagree; 5= strongly agree) |
| **Perceived self-efficacy** | 1. I can prevent skin cancer.  2. I can reduce the time period I work under the sun.  3. I can use a cap while working under the sun.  4. I can use sunscreen while I am working under the sun.  5. In can wear sunglasses while I am working under the sun.  6. I can recognize the signs and symptoms of skin cancer.  7. I can use sunglasses even if my friends make fun of me.  8. It is easy for me to buy a cap.  9. It is easy for me to buy sunglasses.  10. It is easy for me to buy a sunscreen. | 10 items on a 5-point scale (1= strongly disagree; 5= strongly agree) |
| **Protection Motivation** | 1. I have decided to protect my skin more than before.  2. I have decided to reduce my exposure to the sun.  3. I have decided to refer to my physician in case I see any abnormal stain or wart in my skin.  4. I have decided to gain more information regarding my skin health and diseases.  5. I have decided to wear a cap when I am exposed to the sun.  6. I have decided to use a sunscreen when I am exposed to the sun  7. I have decided to wear long-sleeved shirts when I am exposed to the sun for a long time. | 7 items on a 4-point scale (1= not at all ; 4= a lot) |
| **Skin cancer preventive behaviors** | **At present, which of the following measures are taken by you to prevent skin cancer?**  1. Using sunscreen  2. Using a cap  3. Using gloves  4. Using sunglasses  5. Wearing clothes which cover most parts of my body  6. Working in the early mornings and afternoons  7. Referring to a physician in case I see a lesion or wart  8. Skin examination (once every 6 months) | 8 items on a 4-point scale (1= never; 5= always) |
